# Laser MICROSAMPLING of soil microbial community

**DOI:** 10.1186/s13036-018-0117-4

**Published:** 2018-11-28

**Authors:** M. V. Gorlenko, E. A. Chutko, E. S. Churbanova, N. V. Minaev, K. I. Kachesov, L. V. Lysak, S. A. Evlashin, V. S. Cheptsov, A. O. Rybaltovskiy, V. I. Yusupov, V. S. Zhigarkov, G. A. Davydova, B. N. Chichkov, V. N. Bagratashvili

**Affiliations:** 1 Department of General Soil Science, Lomonosov Moscow State University, 119991 Moscow, Russia; 2 Research Center “Crystallography and Photonics” RAS, Institute of Photonic Technologies, 142190, Troitsk, Moscow, Russia; 3 Institute of Nuclear Physics, Lomonosov Moscow State University, 119991 Moscow, Russia; 4 Institute of Theoretical and Experimental Biophysics RAS, 142290, Puschino, Moscow Branch, Russia; 5 Leibniz Universität Hannover and Laser Zentrum Hannover e.V, 30419 Hannover, Germany; 6 Center for Design, Manufacturing & Materials, Skolkovo Institute of Science and Technology, 143026, Skolkovo, Moscow, Russia

**Keywords:** Microbe isolation, Unculturable, Laser cell printing, Biodiversity, Metabolic fingerprinting, *Nonomuraea*

## Abstract

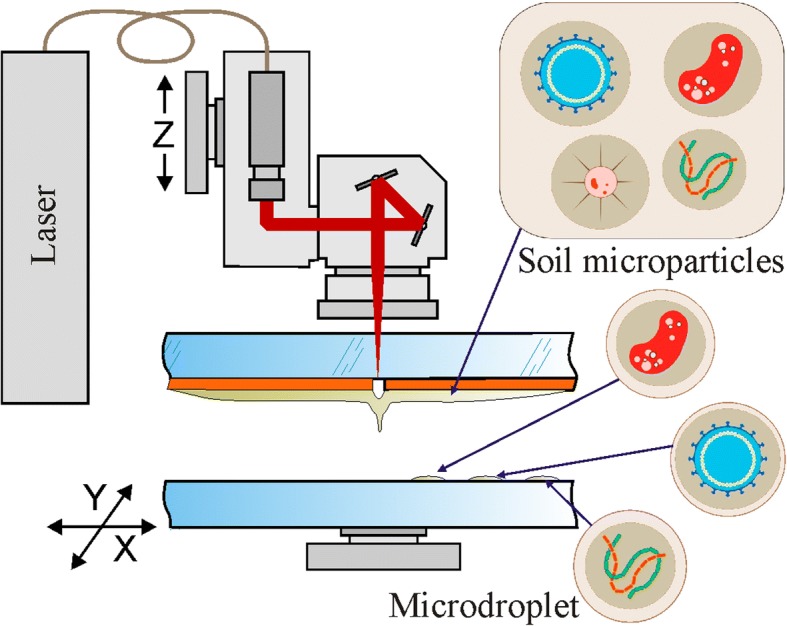

## Introduction

According to recent research, over 90% of bacteria from environmental samples remain uncultivable while using standard cultivation methods on trivial media [1–3]. So-called «yet-to-be-cultured» microorganisms cannot be isolated from native environment using common cultivation procedures [2, 3]. The variety of yet-to-be-cultured bacteria, especially the soil ones, seems to be a largest native prokaryotic gene pool on the Earth. It is remarkably significant for the phylogenetic studies and biotechnological research, in particular in context of new antibiotic producer findings [4].

In bacteriological studies, the standard soil treatment procedures, such as an ultrasonic or vortex processing, imply dispersing and homogenization of a soil sample in liquids that assumes disintegration of soil microaggregates and particles, leads to desorption of microorganisms and destruction of biofilms. The resulting soil slurry is used to spread on a growth nutrient media surface during the standard germ cultivation procedure. The main unculturability phenomena appear due to the following reasons [5, 6]:

- Competition for resources between co-cultivated microorganisms, resulting in rapid development of dominant fast growing strains and in the lack of resources for slow growing microorganisms with chemical inhibition of their growth due to antibiotic production;
- In absence of growth on agar, application of synthetic media not fulfilling the growth requirements for a given organism and/or impossibility for now to select proper artificial media for some highly associated groups, such as symbionts or pathogens;
- Breaking vital liaisons existing in the soil micro microzones peculiar to some organisms, served to sustain the regulation and metabolism.

Some new methods of isolating “yet-to-be-cultured” microorganisms by using simulated natural environment as the Soil Diffusion System have been developed in recent years [7, 8]. A significant progress in the field of cultivation “yet-to-be-cultured” microorganisms has been achieved, by involving cell isolation micro-devices such as hollow fiber membranes [9] and isolation chips (Ichip) [10]. Using these methods, a new super-antibiotic (teixobactin) producer has been discovered [11]. Another new technology for soil microparticle separation, based on laser bioprinting, has been recently demonstrated [12]. In contrast to wide-spread bioprinting processes dealing with a liquid transfer [13–16], soil microparticle printing has been performed in dry or slightly wet conditions (with glycerin addition), resulting in a relatively high degree of microparticle spraying [12].

In the methods of laser bioprinting that has been described earlier, a hydrogel containing living cells and biomolecules is spread on a metal coated donor glass plate. Laser irradiation leads to the metal layer evaporation with a bubble generation [17]. The bubble expansion and collapse results in the formation of a liquid jet [13, 17] transferring a microdroplet containing living cells to the parallel acceptor glass plate. Gel laser bioprinting of eukaryotic cells is characterized by almost 100% cells survival and viability [14]. The viability of bacterial cells after the dry soil printing process still remains unknown [12].

The present work is dedicated to the development of laser soil printing as a powerful laser microsampling (LMS) technique capable to achieve maximum spatial separation of soil microzones, minimizing the microaggregate breakage, and maintaining high cell survival level. Different to the dry soil printing procedure, described in [12], we use a liquid soil/gel mixture to make laser printing of soil micro-aggregates more gentle and precise. This allows preserving original connectionin microbial consortia and reduces the concurrent interactions avoiding some of the major unculturability reasons.

## Results and discussion

### Laser printing of soil microparticles

**Fig. 1:**
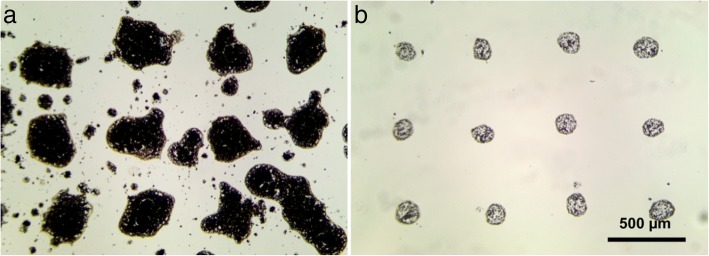
Soil laser micro sampling (LMS) with water (**a**) and with an addition of gel (**b**) produced by laser printing of soil containing drops at the laser pulse energy of 20 μJ

Laser printing of simple water-soil mix (without a gel addition) results in severe spraying, uneven form, and large deviations in the diameter of the transferred droplets (see Fig. 1a). In this figure, printing results of the dense soil slurry are shown. At the same time, printing of droplets containing only single soil particles requires heavy diluted mixes. In this case, single cell printing is possible, allowing separation of soil microbe cells, or printing of a small number of cells in consortia, which can be sufficient for effective suppression of antagonistic influence of the competing cell types, while keeping synergetic bonds. Unfortunately, decreasing the viscosity of the water soil slurry by dilution leads to even stronger spraying and uncontrollable printing results.

Using water-soil slurry mixed with a gel in proportion 1:2 by volume considerably increases viscosity of the mix. Mixed droplets have less spreading on the donor slide and have no spraying (see Fig. 1b). Moreover, addition of gel allows to operate within a wide range of concentrations of soil particles in the mix and to print almost any type of soil.

**Fig. 2:**
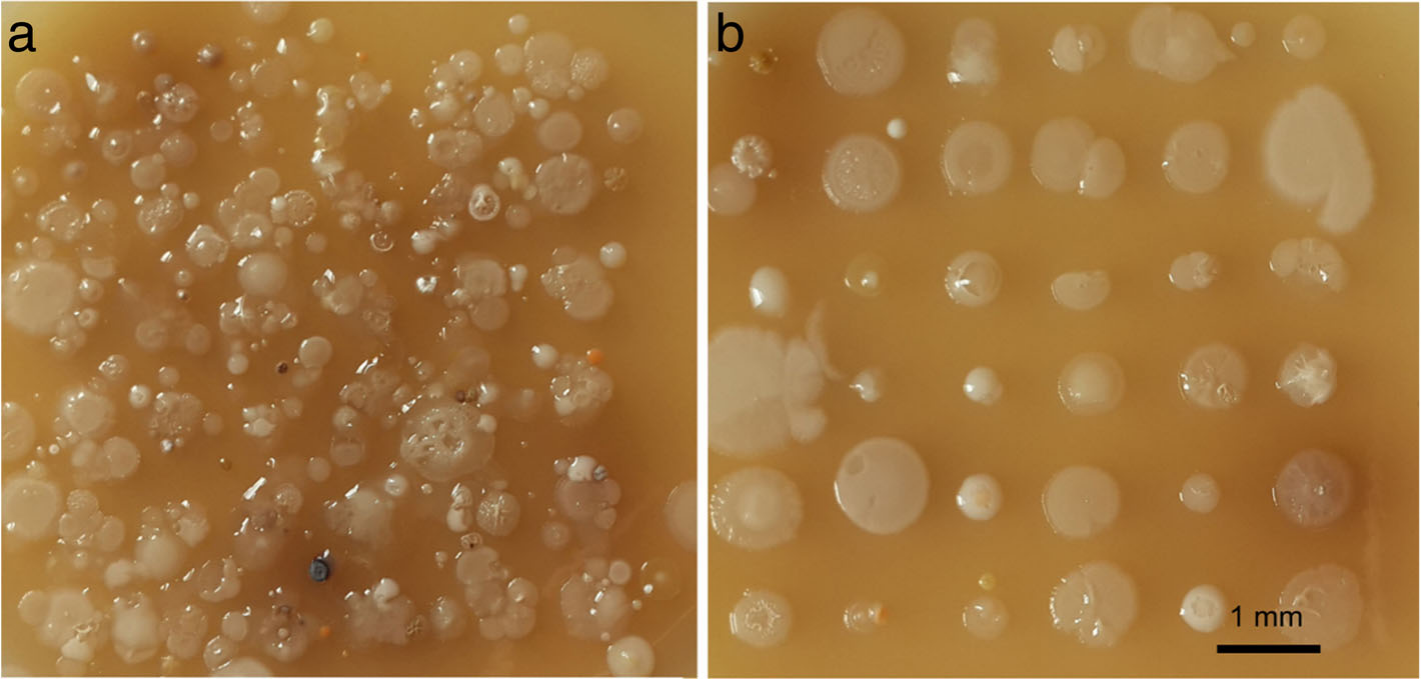
Laser printing of soil microparticle containing 6 × 6 droplet arrays onto agar substrate in cases of soil mix with water (**a**) and with the addition of 2% gel (**b**)

A similar behavior can be observed during the soil printing onto agar plates. After laser printing of soil-water mix without a gel, chaotic growth of colonies with overlapping effects has been observed (see Fig. 2a). In case of gel addition, laser printing of soil microparticles resulted in regular growth patterns of colonies (see Fig. 2b).

### Effects of laser pulse energy, focusing conditions, and viscosity of hydrogel

**Fig. 3:**
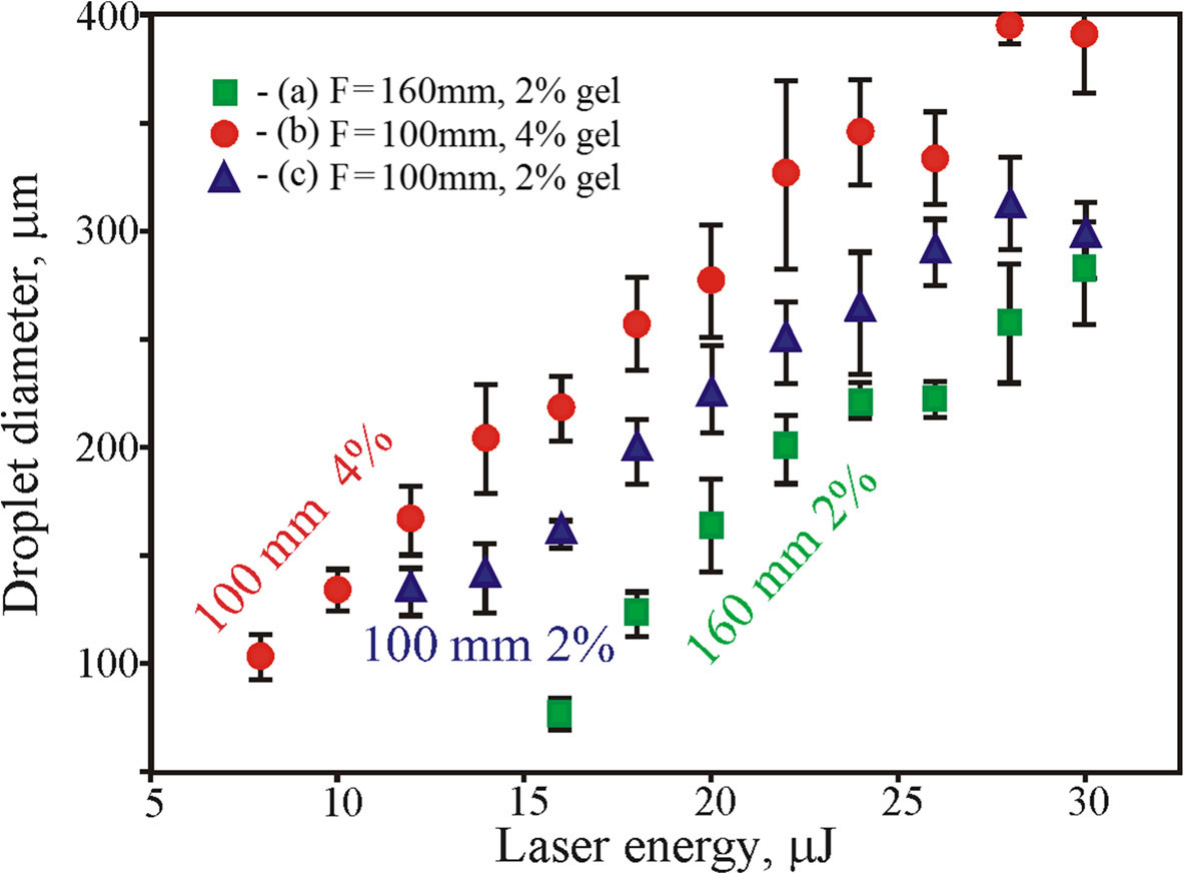
The printed droplet diameter as a function of the laser pulse energy, beam focusing F (focal distance), and gel viscosity: (**a**) - F = 160 mm and 2% gel, (**b**) - F = 100 mm and 4% gel, (**c**) - F = 100 mm, and 2% gel

**Fig. 4:**
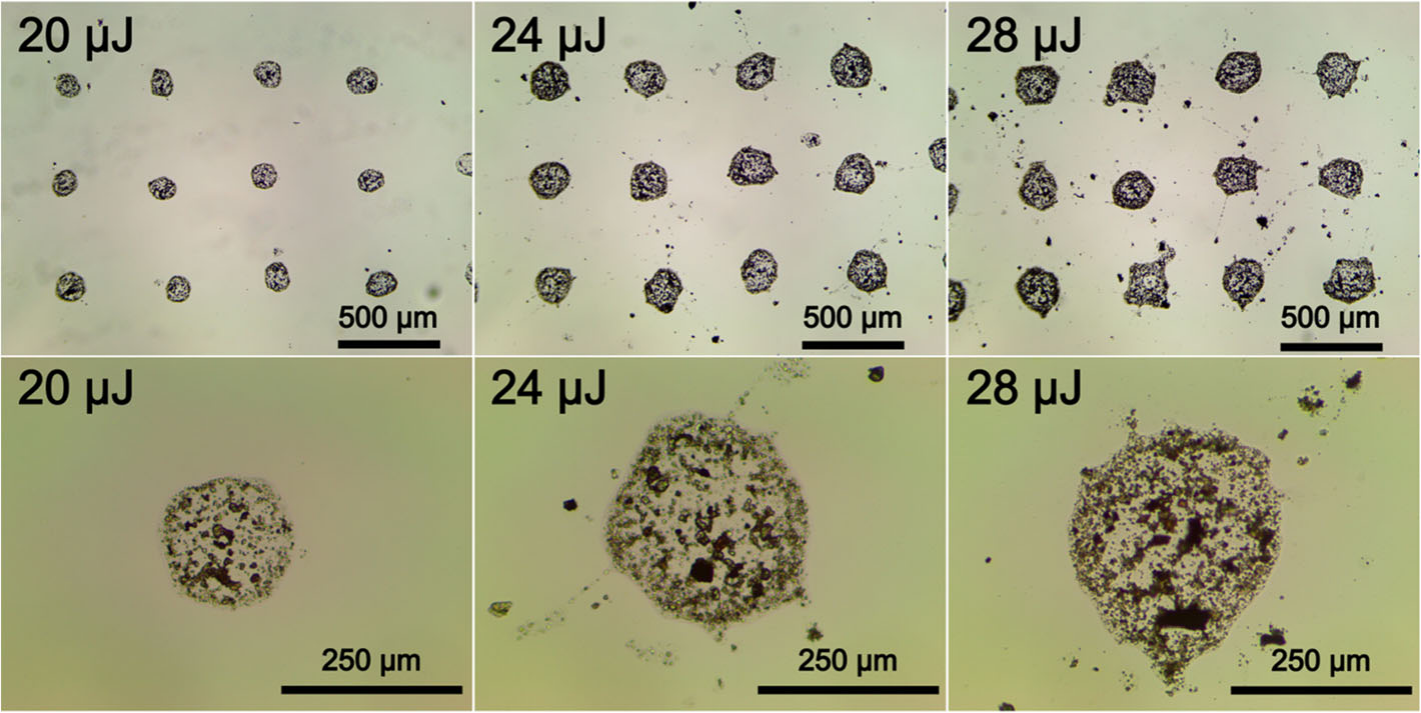
Microphotographs of soil mix drops with 2% gel printed at different laser pulse energies (*F* = 160 mm)

The laser pulse energy and beam focusing conditions have a great influence on the LMS process. At tight focusing of the laser beam, less pulse energy is required for soil droplets transfer (Fig. 4). The value of the threshold energy for a lens with the focal distance *F* = 160 mm is *E*_th_ = 16 μJ (2% gel), and for a lens with *F* = 100 mm it is *E*_th_ = 8 μJ (2% gel). The laser transfer threshold depends also on the gel viscosity; more viscous 4% gel can be transferred at larger energy than less viscous 2% gel. Among different factors presented in Fig. 3, the combination of lens distance F = 160 mm and soil mix with 2% gel has been recognized as optimal. Using the combination of lens with F = 100 mm and soil mix with 2% gel, strong spraying and instability of droplet transfer was observed. While the soil mix with 4% gel has quicker dried out and blade coating on the donor glass plate was less reproducible. Higher laser pulse energies resulted in a bigger drop sizes with a non-rounded shape (see Figs. 3 and 4) and led to droplet spraying.

As it was shown in [15], such behavior can be explained by an increased laser-induced jet speed and violation of its laminarity at high laser pulse energies*.* At high energies, transfer of droplets leads to their enriching with soil particles. In this case, the number of large particles inside the transferred droplets increases together with their average size (Fig. 4).

The volume of droplets with the growing laser pulse energy varied from 200 to 1000 pl, respectively. On the basis of our experimental studies, optimum laser pulse energies for the LMS of soil-gel mix with 2% gel concentration and lens focus *F* = 160 mm are in the range of 20 to 24 μJ.

### Microbial growth in Petri’s dishes on agar plates of a glucose-peptone-yeast agar

#### Experiments with Mollisol

**Fig. 5:**
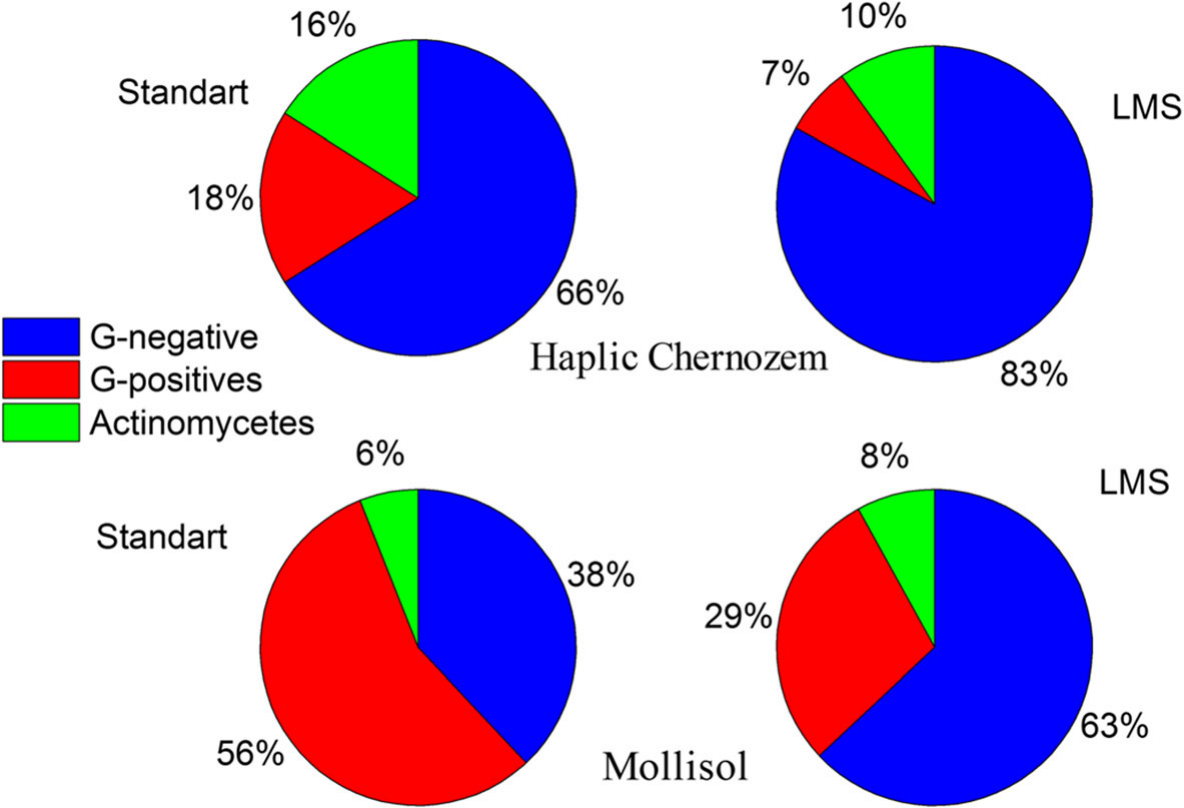
Distributions of groups of microorganisms in cases of standard inoculation method and LMS procedure for Haplic Chernozem and Mollisol

After the LMS procedure, microorganisms were cultivated on a solid surface of nutrient media (glucose-peptone-yeast agar) in Petri dishes. 400 colonies germinated after LMS were compared on genera level with 250 ones grown after the common plating. All of them have been analyzed. We consider that number of colonies analyzed was enough to cover the bacterial diversity (culturable in the conditions used) due to multiple detection of the same morphotypes and genera. Good’s coverage [18] calculated based on genera identification was 99.8 and 100% for LMS and common plating, respectively. All colonies were described and identified on the basis of phenotype analysis and potassium hydroxide test. The percentage distribution of Gram-positive (GP) and Gram-negative (GN) bacteria and actinomycetes was determined (see Fig. 5). Application of the LMS method increased the abundance of GN bacteria from 38 to 63%. It is well-known that the GN bacteria have thinner cell wall compared to that of GP bacteria which explains higher sensitivity of the GN bacteria to external influences. In particular, destruction of some cells at cells desorption procedures (vortexing or ultrasound treatment) can be assumed. The observed bacteria abundance demonstrates that the LMS procedure is friendlier for the GN bacteria compared to the standard inoculation method. In the presence of stronger breaking influences characteristic to the standard soil pre-treatment, dominance of more resistant GP spore forming bacteria is observed.

More detailed taxonomical variety on genera level of cultivated bacteria is presented in Table 1. The standard inoculation procedure allowed cultivating only three genera of GP bacteria namely *Bacillus*, *Arthrobacter*, *Streptomyces* and one GN genera *Myxococcus*, which is able to form microspore with high stress resistance. The LMS procedure leads to an increasing number of GP cultivated genera, up to 8 adding *Paenibacillus*, *Rhodococcus*, *Nocardioides*, *Microbacterium* and *Nonomuraea* which were not found by the standard inoculation method. The number of GN cultivated genera grew up from one to six due to the LMS application. More significant increase in GN bacteria abundance was achieved using sequential inoculation of microbial biomass grown up on monosubstrate liquid media from wells of “Eco-Log©” microplate after the LMS germination to universal solid growth media. This method allowed cultivating of *Xanthomonas*, *Aquaspirillum*, *Spirillum*, *Erwinia* and *Escherichia*. The described improvements in the isolation procedure increased the number of GN isolates up to 11 genera. Because of the substrate composition and base media in “Eco-Log©” plates focused on GN cultivation, no such effects have been observed for GP bacteria.

**Table 1:** A taxonomic variety of bacteria isolated from Mollisol using the standard method of inoculation, LMS procedure, and microplate wells

Taxonomic diversity of bacteria			
Standard inoculation	LMS method	Microplate wells	
Gram-positive (genera):			
Bacillus Arthrobacter Streptomyces	Bacillus Paenibacillus Arthrobacter Streptomyces Rhodococcus Nocardioides Nonomuraea Microbacterium	Bacillus Arthrobacter Rhodococcus	
Gram-negative (genera):			
Myxococcus	Myxococcus Cytophaga Sporocytophaga Pseudomonas Enterobacter Serratia	Myxococcus Cytophaga Sporocytophaga Pseudomonas Enterobacter Serratia	Xanthomonas Aquaspirillum Spirillum Erwinia Escherichia

A special attention should be paid to the 3–1 Str strain (GenBank accession number KY363604), which is related to *Nonomuraea* genus on the basis of phylogenetic identification and was isolated by the LMS method without creation of any unique cultivation conditions. The genus *Nonomuraea* is a rare genus of actinomycetes which isolation by standard methods requires applying of selective media and addition of antibiotics cocktails [19, 20]. It is important that *Nonomuraea* genus members can form a number of bioactive compounds which can be applied for medical, pharmaceutical, and agricultural purposes [19]. In connection with the trends in the search for new metabolites of *Nonomuraea*, it is planned to study the biological activity and to provide proper species identification of the strain isolated by us.

### Experiments with haplic Chernozem

Culturing from this sample was carried out during succession of microbial community initiated by wetting with sterile DI water, due to the fact that a low variety of bacteria was initially cultured from the sample. For this soil we observed about 100 colony morphotypes, germinated after LMS, and about 100 ones after the standard inoculation. Haplic Chernozem sample shown lower culturable biodiversity than Mollisol, but at the same time it contained high proportion of morphotypes difficult for phenotypic identification. Due to this the representatives of each morphotype were identified on genera level using 16S rRNA genes sequencing. Representatives of each genera listed below were found as minimum twice, and Good’s coverage was 100% for LMS and standard inoculation both. During the first 4 weeks, there was no difference in the taxonomic composition of bacteria cultured by standard inoculation and LMS. Representatives of the genera *Arthrobacter*, *Paenibacillus*, *Bacillus*, *Streptomyces*, *Sphingomonas*, *Burkholderia*, *Paracoccus*, *Methylobacterium*, and *Cytophaga* were found. However, in the later stages of succession, a twofold greater variety of bacteria was cultured by LMS compared to standard inoculation. Using LMS, representatives of genera of both GP (*Staphylococcus*, *Microbacterium*) and GN (*Cupriavidus*, *Tardiphaga*) bacteria were found, which previously have not been detected in this sample (Table 2).

**Table 2:** A taxonomic variety of bacteria isolated from Haplic Chernozem using the standard method of inoculation and LMS procedure

Time of succession	Standard inoculation	LMS method	
6th week	Bacillus Streptomyces Burkholderia	Bacillus Streptomyces Staphylococcus	Burkholderia Cupriavidus Tardiphaga
8th week	Streptomyces Burkholderia Bordetella	Streptomyces Bacillus Microbacterium	Burkholderia Cupriavidus Tardiphaga

### Substrate utilization

**Fig. 6:**
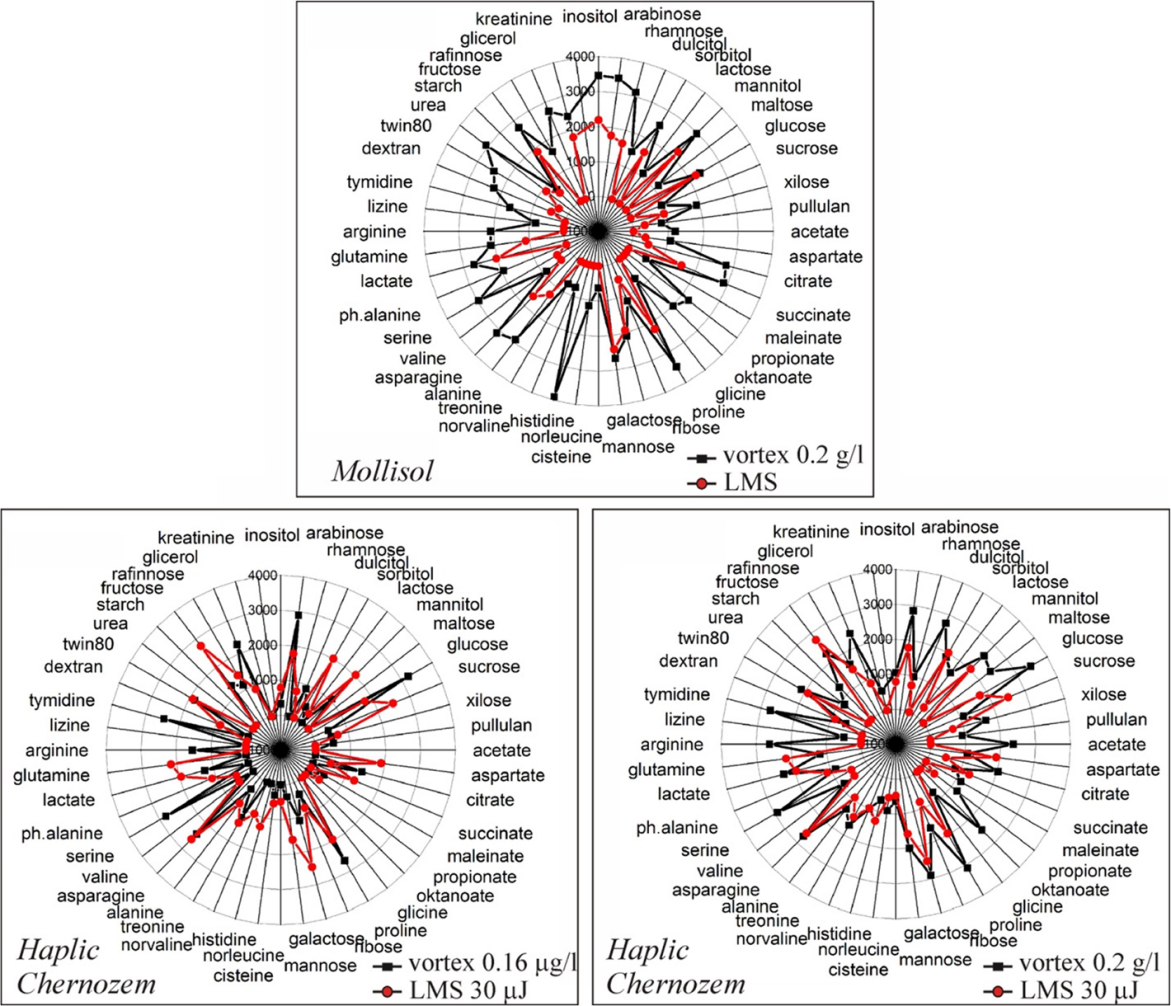
Substrate utilization spectra of soils treated by the standard method and LMS procedure (4 reps averaged)

In Fig. 6, the substrate utilization spectra of Mollisol and Haplic Chernozem microbial communities are presented. These spectra were obtained from “Eco-Log©” microplates (4 reps averaged) inoculated using the standard and LMS methods. In case of Mollisol, the standard inoculation regime was used as a vortexed soil- water suspension in one concentration 0.2 g/l. In further experiments, the Haplic Chernozem in two concentrations was inoculated. We used soil-water-gel mix to equalize conditions in both experimental cases. The highest concentration been used was 0.2 g/l and the lowest one – 0.16 μg/l. In case of using the highest dilution for standard inoculation, the amount of soil transferred in each well of the microplate was approximately equal to the soil amount transferred by microdroplets during the LMS procedure. As demonstrated in Fig. 6, for both soils, substrate utilization patterns obtained by the classic way significantly differ from the spectrum received after the LMS procedure. It is important to note that for both soils on low diluted soil-water suspensions (0.2 g/l) narrowing of the substrate utilization spectra for the LMS samples has been observed. It can be explained by the fact that the LMS procedure, in contrast to the standard technique of soil processing, allows to avoid mixing of microorganisms from different microzones. We select the parameters of the laser transfer in such a way that the effective transferred volume of the gel contains single micro particle of soil. Thus, LMS allows to “cut out” the narrower parts of the microbial community, increasing functional diversity by removing direct competition and separating the substrate niches in microplate wells. We suppose consortium of micro particle carry out the metabolically fingerprint of entire community, that supports “functional doubling” of the system. It means that the functional integrity of the system, supports by switching the units with different sustainability and growth requirements to environment but keep the same functionality meanwhile. We assume our microparticles are such a units. It is possible to assume that the lack of metabolic activity on some substrates occurs due to the declining number of sampled bacteria cells, which is the aim of the LMS procedure. Drastic reduction of the sampled volume, when the droplet volume approaches to 200 pl, plays also an important role. Therefore, the restriction of metabolic diversity, as a result of the LMS procedure, may be considered as a good evidence for selective laser printing of particular microorganisms inhabiting soil microparticles. In contrast to Mollisol, a highly-diluted Haplic Chernozem soil-water-gel mixes produce even broader substrate utilization spectra compared to the standard treatment, which could be explained by a possible laser activation of bacterial growth and metabolism (e.g. [21, 22]).

**Fig. 7:**
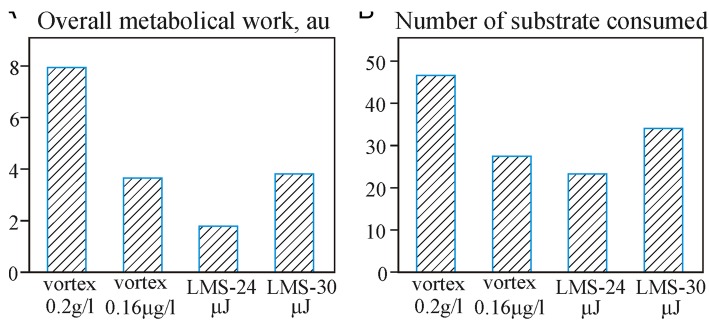
Overall metabolic work (**a**) and functional biodiversity (**b**) of microbial system at different experimental conditions for Haplic Chernozem soil

The analysis of overall metabolic work (presented by the total optical density of plate wells with consumed substrates) and the number of substrates been consumed (metabolic diversity) shows clear differences between the applied experimental regimes (Fig. 7).

It is clear that 30 μJ laser pulses demonstrate the highest performance in biomass transfer. It is easy explainable due to the larger droplet size.

**Fig. 8:**
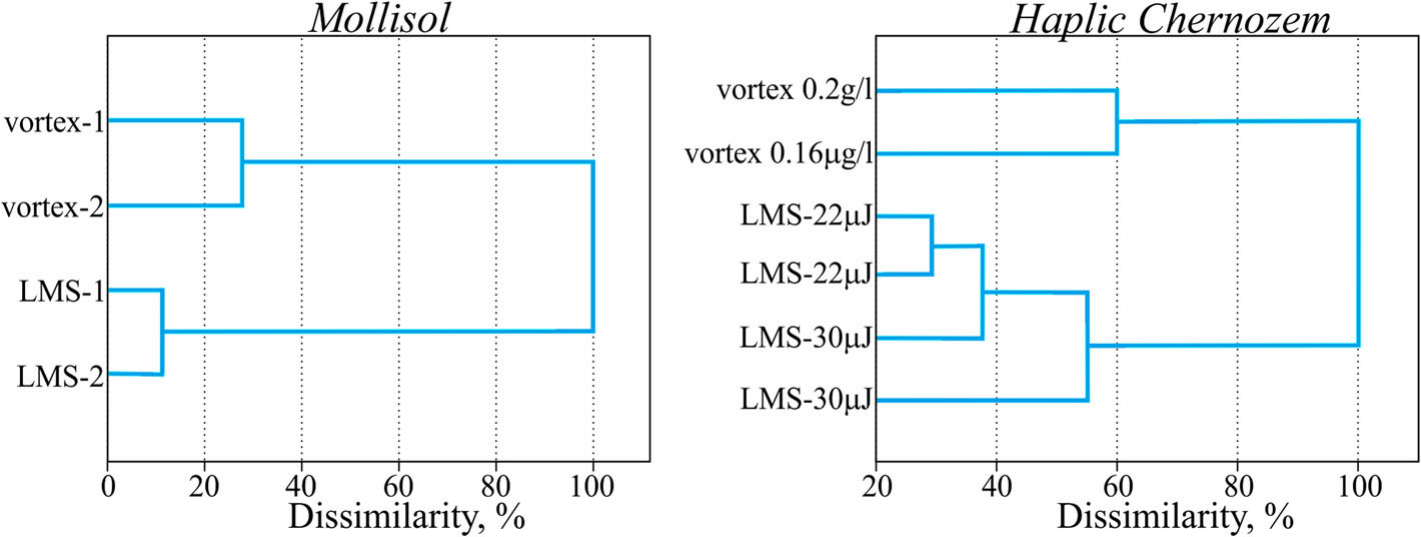
Results of the cluster analysis of substrate utilization patterns after the LMS and ordinary vortex treatment. Ward’s clustering, Euclidean distances. Number of cases: 4 cases of 4 averaged repetitions for Mollisol; 6 cases of 4 averaged repetition for Haplic Chernozem. Number of variables (substrates): 47

Figure 8 demonstrates results of the cluster analysis (Euclidean distance, clustering by Ward) of the presented multidimensional substrate utilization spectra.

By analyzing substrate utilization patterns of Mollisol, we can classify the cases and reveal the significant influences and structural bonds. On the basis of the this dendrogram it is possible to draw a conclusion that the substrate utilization patterns of microbial consortia isolated by the LMS and standard treatments appear to be considerably different. At the same time, convergence of replicas in laser printing is slightly higher (about 10% dissimilarity), than that when the classical method of sample processing (vortexing) is used (30% dissimilarity). It means the increased homogeneity of the LMS samples. It seems LMS procedure allows transferring some “functional diversity quantum” that remains stable in replicas. The application of LMS resulted in reduction of metabolic diversity. The observed reduction means that not the whole community was extracted but only an isolated part. Note that the isolated laser printing of cells and microconsortia is an ultimate goal of the demonstrated LMS method, so it is good evidence in this particular case.

Experiments with Haplic Chernozem demonstrate approximately the same trend. The difference between the substrate utilization patterns produced at different LMS regimes is noticeable. We also have to highlight the fact that in vortex cases, in spite of impressive (10^6^) dilution, substrate utilization patterns remain quite similar. It demonstrates the remarkable stability of the “metabolical fingerprint” of soil microbial community.

### Conclusions

A high throughput Laser Micro-Sampling (LMS) technology for direct isolation from soil of pure microbial cultures and microbial consortia has been developed. This technology is based on laser printing of soil microparticles by focusing near-infrared laser pulses on specially prepared samples of a soil/gel mixture spread onto a gold-coated glass plate. Laser printing occurs in form of direct transfer of soil microparticles to agar surface or into microplate wells.

Different transfer modes of soil microparticle have been described and experimental protocol of laser printing of soil/gel droplets has been optimized. On the basis of tests with the Mollisol and Haplic Chernozem soils, the LMS method has been confirmed to be suitable for printing soil microparticles with maintaining sufficiently high viability of microbial cells. In cultivation experiments, a considerable growth of germinated bacterial diversity after the LMS procedure has been observed. Application of the LMS method significantly increased the number of genus of isolated bacteria, both Gram-negative and Gram-positive groups, which were not cultivated from soil samples with the demonstrated abundance using the classical inoculation method, by putting soil suspensions on the surface of agar plates. A representative of the rare genus *Nonomuraea* was isolated due to the LMS technology without application of any complex selective media and addition of antibiotics cocktails.

Changes in functional diversity and narrowing of substrate utilization spectra induced by the LMS method have been demonstrated in contrast to low diluted systems. The observed decrease in the number of substrates been consumed after the LMS treatment provides an evidence for microbial separating ability of this method. In case of comparison with highly diluted samples, LMS leads to increased metabolic capability of the extracted microbial system that can be explained by possible laser activation. Combined cultivation technique, that includes subsequent solid media cultivation of microbial biomass previously grown on sole carbon substrate media in wells of the LMS inoculated multisubstrate microplate, dramatically increases the taxonomical abundance. This could be a new promising technology to achieve a higher level of rare or yet-to-be-cultured strains isolation. We hope that the LMS method will allow discovering an isolate rare and possibly new species of microorganisms.

In conclusion, we have to point out LMS is goes out of the frames of one narrow task such a soil transfer. It would be successfully used for wide variety of purposes such a bacterial consortia separation. In conclusion, it seems to us that the development of Laser Engineering of Microbial Systems (LEMS) and the study of its possibilities will be promising.

## Materials and methods

### Laser microsampling technique

**Fig. 9:**
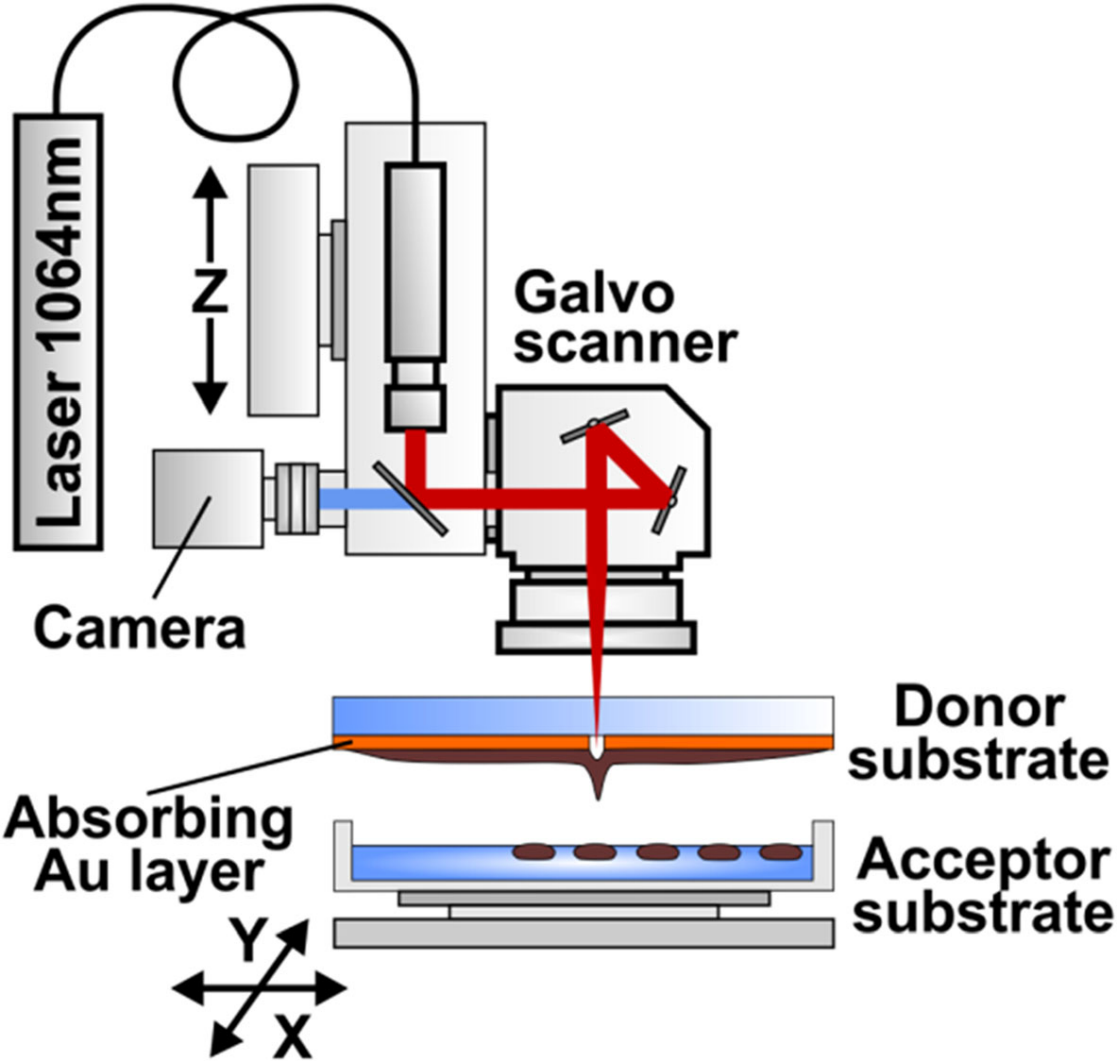
Scheme of the laser microsampling setup

The experimental setup used for printing of soil microparticles is shown in Fig. 9. Radiation of a nanosecond ytterbium fiber laser YLPM-1-4 × 200–20-20 (IRE-Polus, Russia) with parameters λ = 1.06 μm, τ = 8 ns, ν = 105 KHz, E = 10–40 μJ is focused by F-Theta lenses with the focal length F = 100 mm or F = 160 mm (Ronar-Smith, Singapore) onto a gold absorbing layer at the donor glass plate. Laser focus positioning in the target plane has been controlled by the scanning mirrors installed at the lens front. Printing of soil microparticles has been carried out from the donor slide to the collector glass plate located at a distance of 1 mm. The donor glass plate has been coated with a 50 nm thick gold layer covered with a layer of soil mix. Homogeneous coverage with the soil mix has been produced using a blade coater. In our experiments, thickness of the soil mix layer was 30 μm and been regulated with the accuracy of 1 μм by means of a micrometric vertical feed screw motion. Thinner layers (10–15 μm) appeared to be quickly drying out and did not provide enough time to work with the samples. For cultivation of microorganisms and their subsequent studies, laser printing of drops with a soil/gel mix has been carried out directly onto a surface of glucose-peptone-yeast agar in Petri’s dishes, and in 96-well “Eco-log” test plates. The “Eco-log” test plates have been used both as a functional diversity analysis tool and as a set of intermediate selective synthetic sole carbon substrate media.

### Soil mix for laser microsampling

There were two soil samples have been used for soil mix preparation: top 10 cm layer of Mollisol (Belgorod region, Russia) and 19–32 cm layer of Haplic Chernozem (Saratov region, Russia). 0.2 g. of the dry soil has been mixed with 0.4 ml of water and 0.8 ml of hydrogel. The soil/gel mix has been gently homogenized by glass stick. In our experiments, polysaccharide gel based on low-molecular hyaluronic acid (Hyaluronic acid sodium salt from *Streptococcus equi*, Sigma) has been used. A gel addition increases the mix viscosity and reduces spraying of printed drops on the collector surface [23]. However, the gel possibly could be used as a growth substrate by some microorganisms, leading to a partial change of the cultured biodiversity. The gel preparation procedure includes addition of phosphate buffer solution (PBS) to the dry hyaluronic acid in proportions of 2 and 4% following cooling down at + 4 °C for 48 h to reach full dissolution.

The standard soil slurry has been used as a control: 5 g of this soil was mixed with 45 ml of deionized (DI) water and vortexed with subsequent differential centrifugation (1000 rpm for 1 min, 3000 rpm for 3 min) for fractionation; a tiny fraction has been separated and diluted with water to a required consistency. We concede possible to use DI water for soil samples dilution regarding the soil as a substance quite enriched with salts and electrolytes, so we do not expect a significant osmotic shock for Gram-negative (GN) bacteria. The experiments with Haplic Chernozem implied using of soil/gel mixes in standard plating procedure. The Mollisol soil particle size distribution, recorded by Microtrac S3500 Particle Size Analyzer, demonstrated that the most of soil particles have sizes in the range of 5–50 μm. For Haplic Chernozem the most of soil particles has sizes in the range of 0.3–60 *μ*m.

### Microbiological methods

#### Cultivation and identification of bacteria

Direct laser printing on a surface of Petri’s dishes covered by solid glucose-peptone-yeast media [24] was performed to estimate bacteria growth ability after the LMS process. Cultivation with the standard technique (plating using glass spatula) from vortexed soil slurry was also produced to estimate the effect of laser printing [25]. Similar as for LMS method soil dilution was used – soil\gel proportion 1:5. Moreover, for Halphic Chernozem, cultivation by the standard technique from the soil-gel mixture was carried out to avoid possible effects induced by osmotic shock and the gel presence. The dishes were incubated at a temperature of 28 °C for 5–7 days and check the gel influence. There no significant effect of gel at all. The number of grown bacteria colonies was counted with the main morphotypes detection. Identification of bacteria cultures has been carried out on the basis of phenotypical, cultural, micromorphological, and physiology-biochemical signs as key factors for phylogenetic identification of soil bacteria growth [26] and determinants of bacteria manuals [27].

Some strains, which identification using phenotypic characteristics was difficult, were identified by analysis of nucleotide sequences of the 16S rRNA gene. The gDNA extraction was performed using “Probe-Express” kit (“Syntol”, Russia) with adding of 5% Triton X-100 (“AppliChem”, Germany), followed by 10-min boiling at 100 °C, and subsequent treating by glass beads (50–200 μm) using Mini-BeadBeater homogenizer (USA) at 5000 rpm, 60 s. Polymerase chain reactions (PCR) were carried out using “ScreenMix” PCR-kit (“Evrogen”, Russia) with 63f + 1100r and 63f + 537r primers [28, 29] for *Actinobacteria* and with 63f + 1387r primers [28] for other bacteria. PCR-products were purified and sequenced (Sanger dideoxy sequencing) by “Syntol” company (Moscow, Russia) using 537r and 1100r primers [29]. All nucleotide sequences were manually verified using Chromas Lite 2.01 (http://technelysium.com.au/wp/). They were aligned and compared using CLUSTALW2 (https://www.ebi.ac.uk/Tools/msa/clustalw2/) program and closest relatives were found by using the BLASTn (NCBI) sequence search utility (https://blast.ncbi.nlm.nih.gov/). Nucleotide sequences were added to the GenBank under the numbers KY363598-KY363608 and MF445164-MF445186 for strains, isolated from Mollisol and Haplic Chernozem, respectively.

### Multisubstrate testing

The multisubstrate testing (MST) method, based on “Eco-Log©” technology, is a novel method in soil microbiology [30] designed to estimate the functional diversity of microbial communities. It is an our realization of community level physiological profiling assay. The most known wide spread analog - a BIOLOG™ technique [31]. This method allows estimating a capability of microbial community to consume а set of sole- carbone -sources media and obtaining the substrate utilization pattern - multidimensional “metabolic fingerprint” of a microbial system. That represents the ability to grow on different separate organic substrates and is described as microbiological functional diversity of environ. This method is useful for assessing the functional state of microbial communities, which reflects the influence of various external factors, in particular, environmental pollution. In fact, the method allows possible to monitor the ecological situation. [32, 33].

To obtain a substrate utilization pattern, microdroplets of the soil slurry have been transferred directly (by the LMS or standard method) into wells of 96th well test microplate “Eco-Log©” that contains a set of 47 test substrates in two replicates (each well contains one separate substrate, a mineral media, and the indicator of substrate consumption -tetrazolium bromide) (Table 3). The indicator of growth of microorganisms gets purple colored due to the cell respiration. Color intensity is proportional to the number of electrons passing through the membrane during respiration on a given substrate and correlate with the density of biomass grown on a test substrate. Test substrates used in this system include different sugars, amino acids, polymers, nucleosides, salts of organic acids, alcohols, etc. Usually, in data processing two replicas plate data are averaged and treated as a 48-dimentional data vector. 2 plates with 2 reps were used for each experimental point (4 reps totally).

**Table 3:** Sole carbon sources for the Multisubstrate Testing method

Nominal group of substrates	Substrates
Pentoses	Arabinose, ribose, xylose
Hexoses	Glucose, fructose, rhamnose, galactose, mannose
Oligoses	Raffinose, lactose, maltose, sucrose
Salts of carboxylic acids	Acetate, aspartate, citrate, succinate, maleinate, propionate, octanoate, lactate
Amino acids	Glycine, proline, norleucine, histidine, norvaline, treonine, alanine, asparagine, valine, serine, phenylalanine, glutamine, arginine, lysine, cisteine
Alcohols	Dulcitol, glycerol, inositol, sorbitol, mannitol
Polymers	Starch, Dextran 500, Tween 80, pullulan
Amides, Amines, Nucleosides	Creatinine, thymidine, carbamide

After the LMS procedure, microplates were incubated in a thermostat at 28°С for 72 h. At the end of incubation, the photometric reading of stained cell density (at 510 nm wave length) was carried out by hardware and software of the “Eco-Log©” system to estimate the intensity of substrate utilization [34]. A multidimensional data array was generated as a result of photometric measurements of values of optical density on all wells (all substrates). It represents a substrate consumption spectrum of for this soil microbial system -“metabolic fingerprint”. For identification of the microorganisms grown in some wells, the microbial biomass has been transferred from the wells to solid media for subsequent cultivation and identification.

The microplate technology was used for two purposes: 1) to estimate functional diversity of the transferred microbial systems; 2) to use as sole carbon source selective media for precultivation that potentially reveals unusual niches for rare strains. We consider the microplate technology as a model of soil microzones. It can provide advantages for rare/new strains isolation by enlarging the logical space in our experiments.

## Abbreviations

GN: Gram-negative
GP: Gram-positive
LMS: Laser Micro-Sampling
